# Noninvasive Mapping of Striosome-Matrix Imbalance in Early Psychosis

**DOI:** 10.1016/j.bpsgos.2026.100785

**Published:** 2026-07-06

**Authors:** Akila Weerasekera, Jeff L. Waugh, Dost Öngür, Garry P. Nolan, Maria Mody, Fei Du

**Affiliations:** a Psychotic Disorders Division, McLean Hospital, Belmont, Massachusetts; b McLean Imaging Center, McLean Hospital, Belmont, Massachusetts; c Harvard Medical School, Boston, Massachusetts; d Martinos Center for Biomedical Imaging, Massachusetts General Hospital, Charlestown, Massachusetts; e Division of Pediatric Neurology, Department of Pediatrics, University of Texas Southwestern, Dallas, Texas; f Department of Microbiology and Immunology, Stanford University School of Medicine, Stanford, California

**Keywords:** Diffusion MRI, Early psychosis, Matrix, Striatum, Striosome

## Abstract

**Background:**

Early psychosis offers a window to identify core neurobiological disturbances before chronic illness and medication effects. The striatum, particularly its striosome and matrix compartments, plays a key role in dopaminergic and corticostriatal function. Disruption of this organization may underlie psychotic symptoms and cognitive impairment.

**Methods:**

Diffusion magnetic resonance imaging and neuropsychological data from 170 participants from the HCP-EP (Human Connectome Project for Early Psychosis) were analyzed. Using probabilistic diffusion tractography informed by animal tracer studies, the striatum was parcellated into matrix- and striosome-like voxels based on differential connectivity to cortical and subcortical targets. Group differences were assessed using analysis of covariance controlling for age, sex, medication exposure, and intracranial volume. Associations with symptom severity (Positive and Negative Syndrome Scale [PANSS]), depressive symptoms (Montgomery–Åsberg Depression Rating Scale), and cognitive performance were evaluated using covariate-adjusted partial correlations.

**Results:**

Individuals with nonaffective psychosis (NAP) showed significantly larger left matrix-like volume compared with control participants (*q* = .001), with affective psychosis participants demonstrating intermediate values. No significant differences were observed in striosome-like volumes. Spatial analyses revealed posterior and inferior displacement of striosomal centers and increased striosome–matrix separation in NAP. Higher PANSS scores were associated with reduced right striosome-like and increased right matrix-like volumes (*q* < .05), while better cognitive performance correlated with greater striosome-like and lower matrix-like volumes bilaterally.

**Conclusions:**

Early NAP shows matrix expansion and altered striosome-matrix organization, indicating compartment-specific corticostriatal reorganization. These findings highlight a novel, noninvasive marker of striatal imbalance linked to symptoms and cognition within dopamine circuitry.

## Plain Language Summary

In this study, we use advanced brain imaging to examine how 2 key parts of the striatum, called the striosome and matrix, are organized in people with early psychosis. These compartments help regulate dopamine, a chemical linked to symptoms such as hallucinations and motivation problems. We found that the balance between these compartments is altered, particularly in patients with nonaffective psychosis. These changes were also related to symptom severity and cognition. Our findings suggest that early psychosis may involve disruptions in brain organization that could help guide future treatments targeting specific brain circuits.

Early psychosis, encompassing both affective and nonaffective forms, is often conceptualized as a critical window spanning the period around illness onset, including both the clinical high risk phase and the early years following onset. In the current study, we operationally define early psychosis as the first 3 years following symptom onset. In this study, diagnostic group serves as the primary exposure, and striatal compartment measures are the outcomes of interest. During this period, potential confounding influences on these brain-based outcomes such as prolonged medication exposure, chronic illness progression, and cumulative psychosocial burden are minimized (1). This stage offers a critical window to identify core neurobiological disturbances and early intervention targets before chronic changes emerge, with the potential to alter disease trajectory and reduce long-term impact.

Psychotic disorders are broadly classified into affective and nonaffective subtypes based on the presence of mood symptoms (2). Affective psychosis (AP) includes conditions in which psychosis occurs during mood episodes, such as bipolar or major depressive disorder with psychotic features (3,4). In contrast, nonaffective psychosis (NAP) includes schizophrenia spectrum disorders, where psychotic symptoms occur independently of sustained mood disturbance (5). This distinction reflects partially divergent mechanisms, with AP linked to limbic and frontotemporal dysfunction (6,7) and NAP more strongly associated with corticostriatal-dopaminergic circuit abnormalities (8,9).

Psychotic disorders are complex conditions characterized by disturbances in perception, cognition, emotion, and motivation (10). Psychosis is increasingly understood to involve disruptions in neurodevelopmental processes, although it is not formally classified as a neurodevelopmental disorder. Multiple genetic, developmental, and environmental factors converge to disrupt corticostriatal and thalamocortical circuits, pathways critical for reward learning, decision making, and cognitive control (9,11). Structural and functional neuroimaging studies have consistently demonstrated alterations in gray matter volume, cortical thickness, and network connectivity within the fronto-striato-limbic system in schizophrenia and related psychoses (12,13). However, the direction and spatial distribution of these effects are heterogeneous. For example, structural studies have commonly reported widespread cortical gray matter reductions alongside subcortical alterations, while functional studies have often shown increased connectivity or activity within striatal and limbic regions and decreased connectivity in cortical networks (12,13). Diffusion imaging findings further suggest reduced integrity of specific frontostriatal pathways, particularly involving limbic-associated regions such as the nucleus accumbens psychoses (12,13). These patterns may vary across illness stages, with early psychosis often characterized by striatal hyperactivity and dopaminergic dysregulation and later stages associated with more pronounced cortical deficits. Dysregulation of striatal dopamine signaling, particularly within associative and limbic subdivisions, is a hallmark of psychosis and underlies both positive and negative symptom domains (9,14,15).

Within the striatum, 2 interdigitated but neurochemically distinct compartments, the matrix and the striosome, play opposing roles in the regulation of dopaminergic tone and cortical input (16, 17, 18). The intricate organization of striatal compartments and their influence on behavior are only beginning to be understood (19). These compartments cannot be distinguished by conventional magnetic resonance imaging (MRI), but they differ markedly in molecular composition, vascularization, and afferent/efferent connectivity. The striosome directly inhibits dopaminergic neurons in the substantia nigra pars compacta, exerting top-down control over dopamine release, whereas the matrix integrates widespread cortical inputs through the canonical direct and indirect basal ganglia pathways (20,21). Striosome-matrix compartmental organization refers to the relative proportion and spatial arrangement of these 2 compartments, with imbalance reflecting shifts in their relative distribution or spatial segregation rather than isolated alterations within a single compartment. Alterations in striosome-matrix compartmental organization have been reported across a range of neurodegenerative, neurodevelopmental, and psychiatric conditions, but these findings do not point to a single, uniform pattern of dysfunction. Instead, they suggest that the striosome-matrix system represents a flexible corticostriatal architecture that can be perturbed in disorder-specific ways. For example, in Huntington’s disease, early degeneration of matrix neurons relative to striosomes is associated with motor disinhibition and affective symptoms (17,22), whereas in autism spectrum disorder, studies have reported expanded striosomal compartments and altered striosome-matrix balance alongside disrupted dopaminergic signaling (23, 24, 25). In substance use disorders, chronic stimulant exposure preferentially engages striosomal circuitry, biasing reinforcement learning toward drug-seeking behaviors (26,27), while in mood disorders, chronic stress has been shown to modulate striosome-dominated microcircuits involved in emotion regulation and decision making (28,29). Taken together, these observations indicate that dysfunction of the striosome-matrix system does not map onto a simple cognitive-versus-affective dichotomy but instead reflects context-dependent alterations within a shared circuit architecture that supports motivation, learning, and behavioral control. Importantly, a striosome-matrix framework provides a systems-level lens through which established dopaminergic and corticostriatal abnormalities in psychosis can be understood by capturing how limbic and sensorimotor influences are differentially organized within the striatum.

Evidence from preclinical and human studies supports compartment-specific striatal dysfunction in psychosis, with antipsychotics showing differential effects across compartments (30,31). Stress, dopaminergic stimulation, and neurodevelopmental factors may differentially impact striosome and matrix circuits, influencing reinforcement learning, emotion regulation, and executive function (28,32, 33, 34). Preclinical work shows that typical antipsychotics preferentially activate the matrix, whereas atypicals favor striosomes, while postmortem studies report altered synaptic density in both compartments in schizophrenia (34). Together, these findings highlight the matrix-striosome architecture as a framework for understanding early circuit-level dysfunction. Compartmental alterations may represent early markers of dysregulated corticostriatal signaling preceding dopaminergic imbalance. Therefore, studying these dynamics in early psychosis may provide insight into disease mechanisms before chronic illness effects. In this study, we aimed to determine 1) whether compartment-specific differences in striatal volume and microstructure exist in early psychosis, 2) how these compartments are spatially organized, 3) whether abnormalities differ between AP and NAP, and 4) how these alterations relate to clinical measures.

To address these aims, we applied a probabilistic diffusion tractography method [Waugh *et al.* (35)] to parcellate the striatum into matrix-like and striosome-like voxels based on differential corticostriatal connectivity. This approach provides an in vivo proxy for compartmental organization informed by known connectivity differences between compartments.

## Methods and Materials

### Data Collection

No data collection with human subjects took place at the authors’ institutions in this study. Structural-diffusion MRI, neuropsychological test measures, and demographic data were downloaded from the HCP-EP (Human Connectome Project for Early-Psychosis) (https://www.humanconnectome.org/study/human-connectome-project-for-early-psychosis), a publicly available image repository. All participants provided written informed consent and were scanned according to procedures approved by the local institutional review board at each participating institution.

### Participants and Study Design

A total of 189 imaging datasets, including 57 healthy control participants and 132 patients, were downloaded from the National Institute of Mental Health Data Archive HCPEP11AllData package. These datasets represented the imaging data available in the imagingcollection01 folder at the time of download. Although the accompanying participant-level file listed 253 participants, imaging datasets were not available for all listed participants, including 11 healthy control participants, 30 participants with AP, and 30 participants with NAP. Datasets were excluded if they were incomplete or failed quality control, including missing diffusion MRI or T1-weighted imaging data, insufficient MRI quality, missing demographic or clinical variables required for covariate adjustment, excessive motion, image artifacts, or FSL/FreeSurfer preprocessing failure. Of the 189 downloaded imaging datasets, 19 were excluded after quality control and data-completeness review, including 2 healthy control participants, 1 participant with AP, and 16 participants with NAP. Only participants with complete imaging, demographic, and clinical data were included to ensure consistency across analyses. Because exclusions often reflected overlapping technical and data-completeness factors, they are reported collectively. Following these criteria, 55 healthy control participants ages 17 to 36 years and 115 patients with early psychosis ages 17 to 35 years, including 27 participants with AP and 88 participants with NAP, were retained for analysis (Table 1). To address potential selection bias, available baseline demographic and clinical characteristics (Tables 2 and 3) were compared between included participants and excluded datasets, and no meaningful differences were observed across key variables (Table S1).

**Table 1:** Demographic Characteristics of Study Participants

Variable	Control, *n* = 55	Patient, *n* = 115	AP, *n* = 27	NAP, *n* = 88	*p* Value, Control vs. Patient^a^
Age, Years	24.8 ± 4.0	22.7 ± 3.7	24.6 ± 4.3	22.1 ± 3.3	<.001
Sex, Female/Male	19/36	45/70	18/9	27/61	.56
Handedness, Right/Left	46/9	99/10	25/2	74/8	.18
Full Scale IQ^b^	115.9 ± 10.9	102.2 ± 17.2	108.6 ± 15.9	100.3 ± 17.2	<.001

Values are presented as *n* or mean ± SD.

a *p* Values were derived from independent-samples *t* tests (age) and χ^2^ tests (sex, handedness) comparing control vs. patient (all).

b Wechsler Abbreviated Scale of Intelligence II.

**Table 2:** Clinical Measures by Phenotype Group

Variable	Affective Psychosis, *n* = 27	Nonaffective Psychosis, *n* = 88	*p* Value^a^
PANSS Total	70.9 ± 15.4	83.7 ± 17.1	<.001
MADRS Total	18.6 ± 9.8	9.4 ± 7.5	<.001

Values are presented as mean ± SD.

MADRS, Montgomery–Åsberg Depression Rating Scale; PANSS, Positive and Negative Syndrome Scale.

a *p* Values reflect between-group comparisons (independent-samples *t* tests) between the affective and nonaffective psychosis groups.

**Table 3:** Medication Characteristics of Patient Groups

Variable	Affective Psychosis, *n* = 27	Nonaffective Psychosis, *n* = 88	*p* Value^a^
Lifetime Antipsychotic Treatment, Months	10.0 ± 16.0	15.3 ± 14.9	.13
CPZ Equivalent Dose, mg/Day	48.1 ± 134.1	190.2 ± 234.5	<.001

CPZ, chlorpromazine.

a *p* Values reflect between-group comparisons (independent-samples *t* tests) between affective and nonaffective psychosis groups.

### Imaging Data

Imaging data were acquired across multiple sites (Boston, Massachusetts and Indianapolis, Indiana) using harmonized 3T Siemens Prisma MRI protocols, including high-resolution T1-weighted structural and diffusion-weighted imaging; detailed acquisition parameters are provided in the Supplement.

### Striosome-Matrix Parcellation

**Figure 1:**
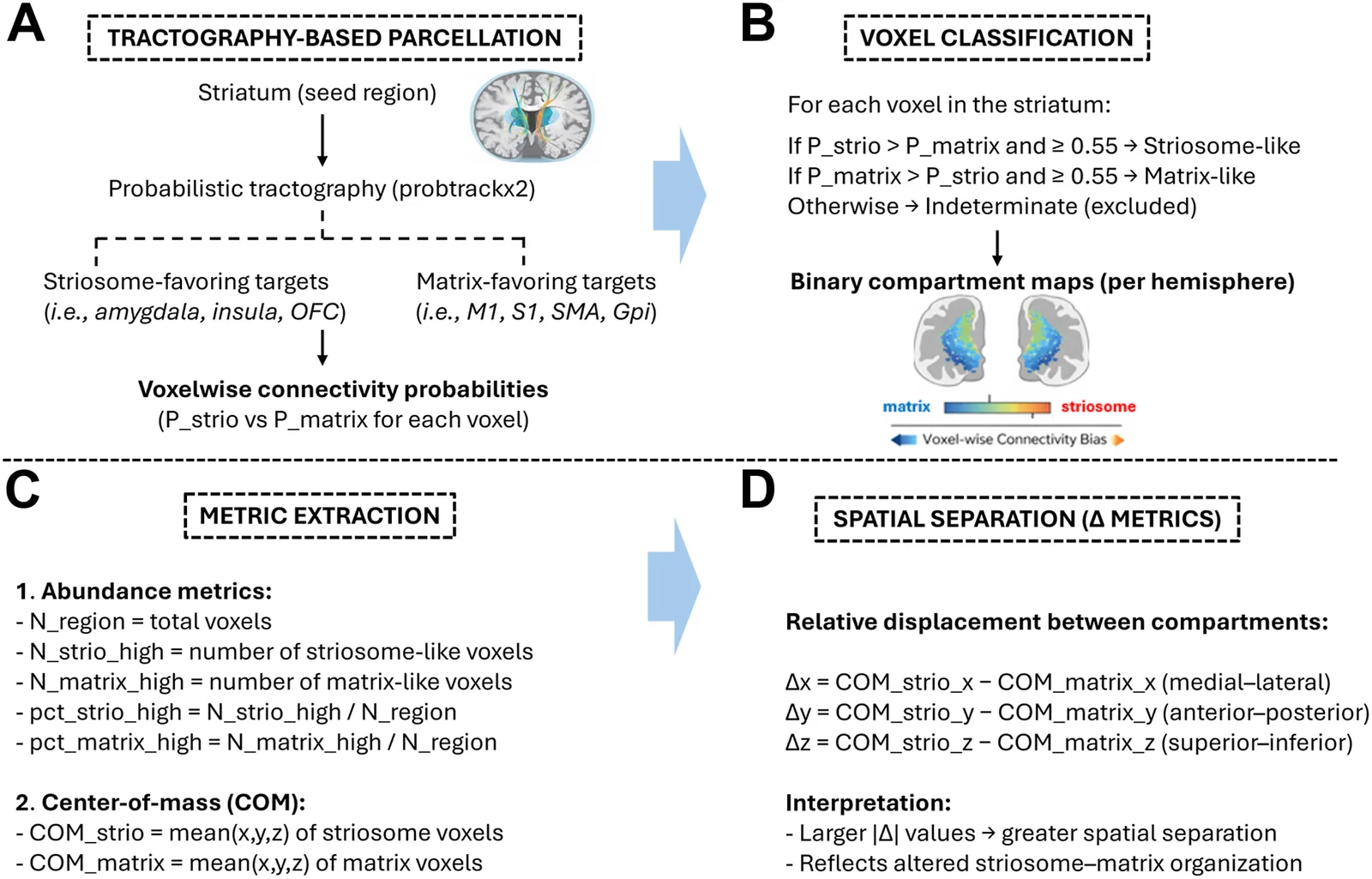
Workflow for derivation of striosome-matrix spatial metrics. Probabilistic diffusion tractography was performed from the striatum to predefined striosome- and matrix-favoring targets to generate voxelwise connectivity probability maps. Voxels were classified as striosome-like or matrix-like based on relative connectivity bias (threshold ≥0.55). Compartment-specific voxel counts and centers of mass (COMs) were then computed for each hemisphere. Spatial separation between compartments was quantified using Δ metrics (Δx, Δy, Δz), representing displacement between striosome and matrix COMs along anatomical axes. GPi, globus pallidus internus; M1, primary motor cortex; OFC, orbitofrontal cortex; S1, primary somatosensory cortex; SMA, supplementary motor area.

**Figure 2:**
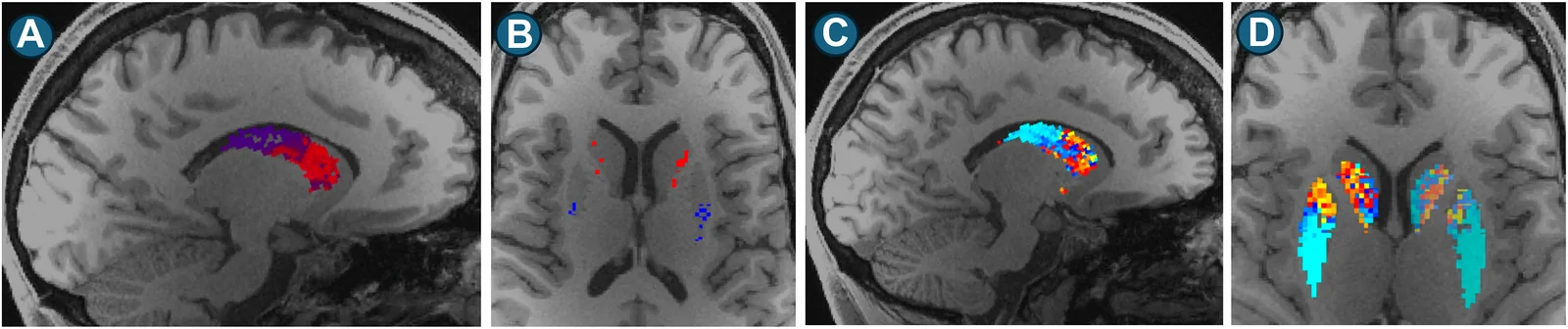
Anatomical distribution of matrix-like and striosome-like voxels. **(A)** A sagittal view (left) of the human striatum showing average classification targets tractography results of the control group, illustrating the probability of connections with matrix-like (blue-light blue) or striosome-like (red-yellow) cortical targets. **(B)** A single participant’s striatum demonstrates the distinct spatial distribution of matrix-like and striosome-like voxels. **(C, D)** Sagittal and coronal views from one individual show the tractography-based probability distribution within the same participant. The right hemisphere is displayed with a threshold of 0.5 to 1, where all voxels include a probability estimate, illustrating the overall spatial organization of matrix and striosome compartments. In contrast, the left hemisphere is displayed with a threshold of 0.75 to 1, masking intermediate-bias voxels. All probability maps are overlaid on the participant’s native T1-weighted anatomical image.

Given the small, interdigitated organization of striosome and matrix compartments, we used a connectivity-based diffusion MRI parcellation approach rather than direct voxelwise anatomical segmentation. This method leverages established differences in corticostriatal connectivity to identify striosome- and matrix-like regions in vivo. Briefly, probabilistic tractography was performed using hemisphere-specific striatal seed masks derived from participant-level anatomical segmentations, with composite striosome- and matrix-favoring cortical and subcortical targets used for classification. Voxels were assigned to compartments based on relative connectivity bias, and equal-volume high-confidence masks were generated to ensure robust and comparable quantification across compartments. This approach provides a biologically informed proxy for striatal compartmental organization while minimizing sensitivity to voxel-level noise and partial volume effects. Complete methodological details, including target definition, tractography parameters, thresholding strategy, mask construction procedures, and ComBat harmonization (Figure 1, Figure 2 and 2), are provided in the Supplement.

### Statistical Analyses

Group differences were assessed using analysis of covariance (ANCOVA) models with group as the main factor and age, sex, and estimated total intracranial volume (eTIV) as covariates, with multiple comparisons controlled using the Benjamini-Hochberg false discovery rate (FDR) procedure; complete statistical details are provided in the Supplement.

## Results

### Caudate and Putamen Volumes

ANCOVA models revealed no significant group differences in either caudate or putamen volumes across the control, AP, and NAP groups (all *p*s > .4). Specifically, group effects were nonsignificant for the left caudate (*F*_2,160_ = 0.37, *p* = .69), right caudate (*F*_2,160_ = 0.43, *p* = .65), left putamen (*F*_2,160_ = 0.92, *p* = .40), and right putamen (*F*_2,160_ = 0.89, *p* = .41).

### Proportion of Striosome-Like and Matrix-Like Volume

**Figure 3:**
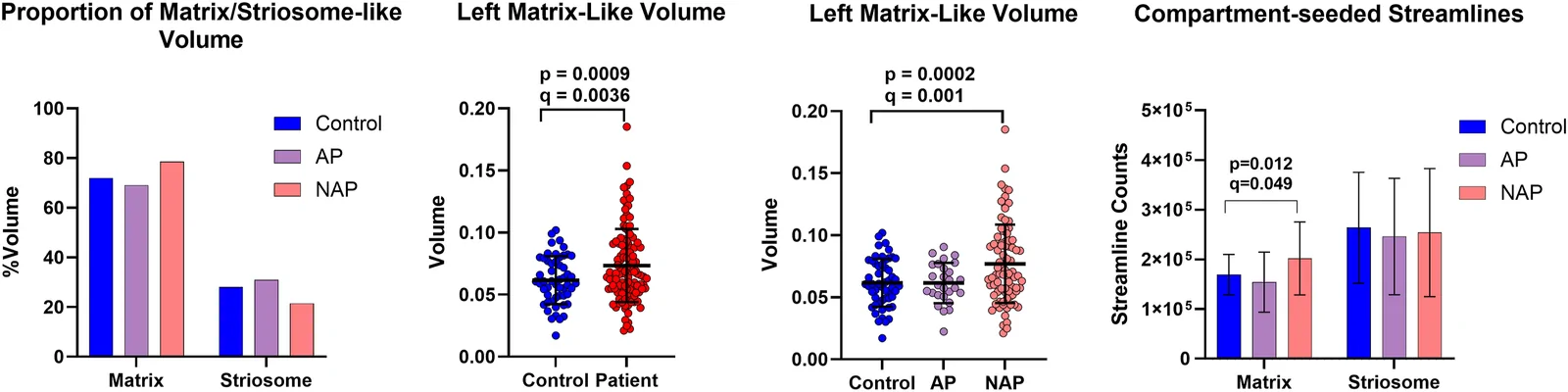
Group differences in striosome-matrix compartment volumes and connectivity. Left: Proportion of matrix- and striosome-like tissue. Mean percentage volumes of matrix- and striosome-like compartments are shown for control participants, participants with affective psychosis (AP), and participants with nonaffective psychosis (NAP). Participants with NAP exhibited an increased proportion of matrix-like volume and reduced striosome-like volume relative to control participants. Middle: Left matrix-like volume differences. Individual data points with mean ± SEM are shown for each group. Compared with control participants, patients demonstrated significantly greater left matrix-like volume (*q* = .0036). This effect remained significant when patients were subdivided into AP and NAP groups (*q* = .001), driven primarily by the NAP group. Right: Compartment-seeded streamline counts. Mean ± SEM streamline counts originating from matrix- and striosome-like seed volumes are shown. Matrix-seeded streamlines were significantly increased in patients with NAP compared with control participants (*p* = .012, *q* = .049), with no significant difference in striosome-seeded streamlines.

Across all participants, the matrix-like compartment constituted the majority of striatum volume, accounting for approximately 74% (±14%) of total tissue, while the striosome-like compartment represented the remaining 26% (±14%). When examined by diagnostic subgroup, the NAP group exhibited the highest matrix proportion (mean ± SD = 75.2% ± 14.8%), followed by the control group (mean ± SD = 72.9% ± 13.5%) and the AP group (mean ± SD = 70.2% ± 11.5%) (Figure 3). Correspondingly, striosome-like proportions were inversely related, ranging from 24.8% in NAP to 29.8% in AP (Figure 3). Although between-group differences in percentage composition were modest, the pattern mirrored the volumetric analyses, with participants with NAP showing a relative expansion of matrix-like tissue and participants with AP showing a slightly higher striosome contribution. These findings may suggest subtle shifts in compartmental organization that parallel absolute volume differences, with the matrix compartment dominating (∼70%–75%) across all groups.

### Volumetric Analysis: Control Participants Versus All Patients

A significant diagnostic effect was observed for the left matrix-like compartment (*F*_1,160_ = 11.44, *p* = .0009, *q* = .0036). Patients exhibited larger adjusted left matrix-like volumes (mean = 575 mm^3^, 95% CI [512–639]) compared with control participants (mean = 478 mm^3^, 95% CI [395–560]). No significant group differences were found for the left striosome or the right matrix-like or right striosome-like compartments (all *q*s > .1) (Figure 3).

### Volumetric Analysis: Control Versus AP Versus NAP

A significant overall group effect was observed for the left matrix-like compartment (*F*_2,160_ = 8.73, *p* = .00025, *q* = .001). Mean left matrix-like volumes were highest in the NAP group, followed by the AP group, and they were lowest in the control group (mean ± SD: control = 0.062 ± 0.019 mm^3^, AP = 0.061 ± 0.016 mm^3^, NAP = 0.077 ± 0.031 mm^3^). Post hoc pairwise comparisons revealed that the NAP group had significantly larger left matrix-like volumes compared with the control group (mean difference = 144 mm^3^, *p* = .0006, 95% CI [54–234]). In contrast, the AP group did not differ significantly from the control group (*p* = .69) or the NAP group (*p* = .09). A trend-level effect was observed in the left striosome-like compartment (*F*_2,160_ = 2.60, *p* = .077), along with marginal effects in the right matrix-like (*F*_2,160_ = 3.24, *p* = .042) and right striosome-like (*F*_2,160_ = 3.40, *p* = .036) compartments; however, these did not survive FDR correction (all *q*s > .1) (Figure 3). Together, these results indicate a selective enlargement of the left matrix-like compartment in the NAP group, with no significant differences involving the AP group or striosome-like compartments.

### Striatal Compartment Streamline Voxel Counts

Group comparisons of striatal compartment diffusion metrics (matrix- and striosome-like mean total streamline counts) revealed a significant group effect for the right matrix compartment (*F*_2,167_ = 4.51, *p* = .012, *q* = .049) (Figure 3). Post hoc contrasts indicated that participants with NAP exhibited higher right matrix streamline means than control participants, whereas the participants with AP showed intermediate values. The left matrix compartment demonstrated a trend-level effect (*F*_2,167_ = 3.04, *p* = .051, *q* = .10). No significant differences were observed for left or right striosome mean values (all *p*s > .33). When analyses were restricted to patient versus control comparisons, none of the matrix or striosome metrics reached significance.

### Microstructural Diffusion Metrics

No significant differences in fractional anisotropy or mean diffusivity (MD) were observed between patients and control participants after covariate adjustment (all *p*s > .4). A trend toward higher MD in patient matrix compartments (*p* ≈ .075) did not survive correction. Overall, microstructural diffusion metrics did not distinguish groups despite volumetric differences.

### Striosome-Matrix Bias Spatial Geometric Metrics

When comparing all patients with control participants, several striatal bias metrics survived FDR correction (*q* < .05). Cohen’s *d* values for these effects ranged from ≈0.3 to 0.5, indicating small-to-moderate increases in the magnitude or spatial separation of striosome- and matrix-like compartments. In the right hemisphere, group effects were weaker (*p* > .2, *d* < 0.3). Overall, these results suggest subtle left-lateralized alterations in compartment organization in patients, although the effects were not significant after correction.

**Figure 4:**
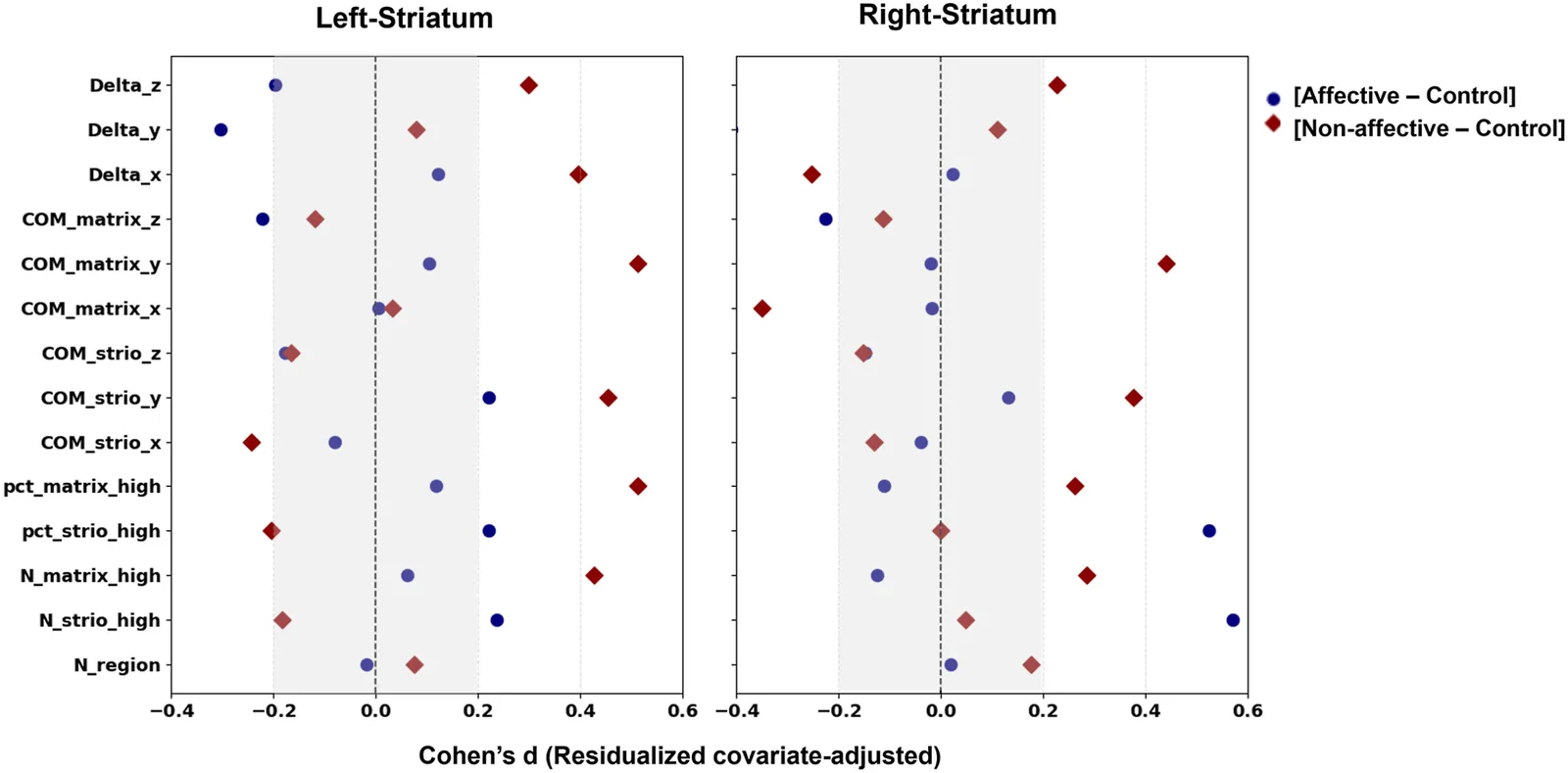
Standardized group differences in striosome-matrix metrics (Cohen’s *d*). Plots display standardized effect size (Cohen’s *d*) for a specific metric, comparing affective psychosis vs. control (blue) and nonaffective psychosis (NAP) vs. control (dark red) across multiple striosome-matrix measures (voxel counts, compartment percentages, centroid positions, and spatial separations Δx–Δz) plotted separately for the left and right striatum. Positive values indicate greater mean values in patient groups relative to the control group. Vertical reference lines at *d* = 0 denote no difference, and shaded regions around 0 indicate small-effect boundaries. Effect sizes are shown separately for the left and right striatum, with larger bars denoting stronger group differences. The legend summarizes the direction and magnitude of effects, highlighting that participants with NAP exhibit the largest deviations relative to control participants across several compartments. COM, center of mass.

A 3-group ANCOVA (control, AP, NAP) revealed no FDR-significant effects, but uncorrected comparisons indicated a similar lateralized pattern. In the left striatum, the NAP group showed higher mean striosome-matrix separation (Δx − Δz) and greater matrix-voxel abundance compared with both the control group and the AP group (*p* ≈ .05–.10, *d* ≈ 0.3–0.5) (Figure 4, left). The AP group generally displayed intermediate values. In the right hemisphere, effects were minimal and nonsignificant (*p* > .3, *d* < 0.3) (Figure 4, right). Together, these findings point to a potential increase in striosome-matrix differentiation in NAP, primarily within the left striatum, although no effects survived correction for multiple comparisons.

**Figure 5:**
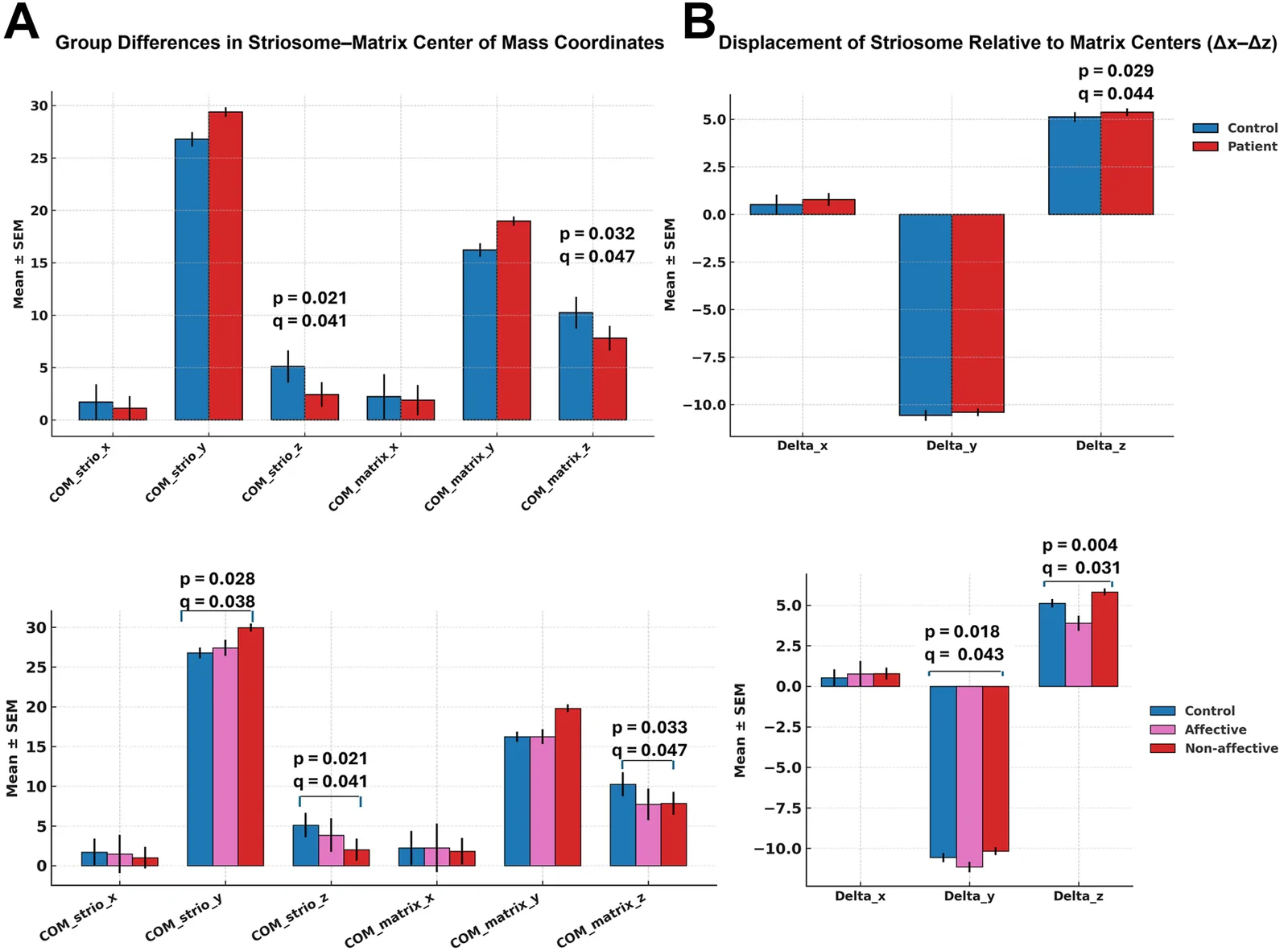
Group differences in striosome-matrix center of mass (COM) positions and spatial displacements within the striatum. **(A)** Bar plots show mean ± SEM values for COM coordinates (left panel) and **(B)** relative displacements (right panel) across control (blue), affective psychosis (pink), and nonaffective psychosis (NAP) (red) groups. COM metrics represent the spatial centers of mass for striosome- and matrix-like compartments in the x (medial-lateral), y (anterior-posterior), and z (inferior-superior) axes. Δx–Δz values quantify the relative displacement between striosome and matrix compartment centers along each dimension. Across both hemispheres, participants in the NAP group showed the largest posterior and superior shifts in striosome-matrix spatial separation compared with control participants, whereas participants with AP exhibited intermediate patterns.

Furthermore, significant group effects in the spatial positioning of striosome and matrix compartments were observed within the striatum. No group differences emerged for voxel counts (*N_strio_high, N_matrix_high*) or proportional occupancy (*pct_strio_high*, *pct_matrix_high*), defined as the fraction of striatal voxels classified as striosome-like or matrix-like, indicating that overall compartment size and relative abundance remained stable across groups. In contrast, centers of mass (COMs) for both compartments showed altered localization. The *COM_strio_z* (superior-inferior axis) and *COM_matrix_z* coordinates differed significantly between groups (*F*_2,__≈160_ ≈ 5–6, FDR *p* < .05) (Figure 4, top row). Post hoc Tukey tests showed that the NAP group exhibited inferior displacement of both striosome and matrix centers relative to the control group (*p* = .021 and .033, respectively), whereas the AP group did not differ from the control group (*p* > .4) (Figure 5, bottom row). Striosome centers were located at z = −1.82 ± 0.09 mm (control), −1.94 ± 0.11 mm (AP), and −2.25 ± 0.10 mm (NAP), indicating a downward shift of ≈0.43 mm in NAP cases. Similarly, *COM_strio_y* (anterior-posterior axis) showed a posterior shift (*q* = .038) driven by the NAP group (mean ± SD = 1.96 ± 0.10 mm) relative to the control group (1.63 ± 0.08 mm, *p* = .028). Matrix positions remained stable along the anterior-posterior direction, suggesting that the striosomal compartment primarily drives these spatial deviations.

**Figure 6:**
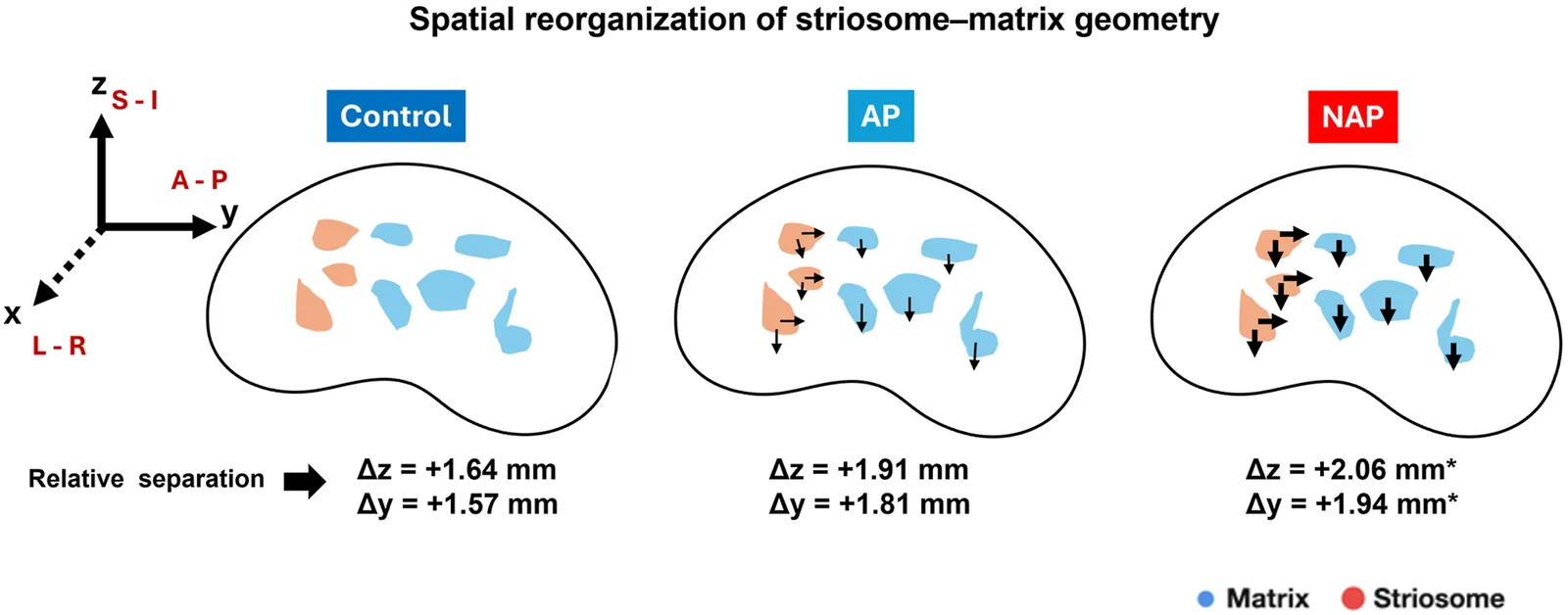
Schematic illustration of altered striosome-matrix alignment across groups. Simplified striatal outlines depict the relative spatial positioning of bilateral matrix (blue) and striosome (red) compartments across control, affective psychosis (AP), and nonaffective psychosis (NAP) groups. The coordinate axes indicate orientation in anatomical space: left to right (L-R) (x), anterior-posterior (A-P) (y), and superior-inferior (S-I) (z). Mean displacements (Δy, Δz in mm) reflect the relative position of striosomal centers of mass compared with matrix centers, averaged across hemispheres. Both patient groups exhibited posterior (Δy) and inferior (Δz) shifts relative to the control group, with the greatest displacements being observed in the NAP group. Arrows indicate the direction of increased separation between striosome and matrix compartments in NAP, consistent with disrupted striatal spatial organization. The size of arrows depicts magnitude of the separation. ∗*p* < .05 vs. healthy control participants.

Consistent with these patterns, relative striosome–matrix displacements (Δ metrics) were significantly elevated along both the Δy (anterior-posterior) and Δz (superior-inferior) axes (*q* = .043 and .031, respectively). Mean separations were Δy = 1.57 ± 0.08 mm (control), 1.81 ± 0.10 mm (AP), and 1.94 ± 0.11 mm (NAP) and Δz = 1.64 ± 0.07 mm (control), 1.91 ± 0.08 mm (AP), and 2.06 ± 0.09 mm (NAP). Post hoc comparisons confirmed greater anterior-posterior and vertical separation in participants with NAP relative to control participants (*p* = .018 and .04), with participants in the AP group showing intermediate, nonsignificant values (Figure 6). Overall, these findings demonstrate that while overall striosome-matrix abundance remains constant, the geometry of the striatal microstructure differs across clinical subtypes. Specifically, patients with NAP show posterior and inferior displacement of striosomal centers and increased striosome-matrix separation, suggesting localized disruption of striatal compartmental alignment in this subgroup.

### Correlations Between Positive and Negative Syndrome Scale Scores and Striatal Compartment Volumes

**Figure 7:**
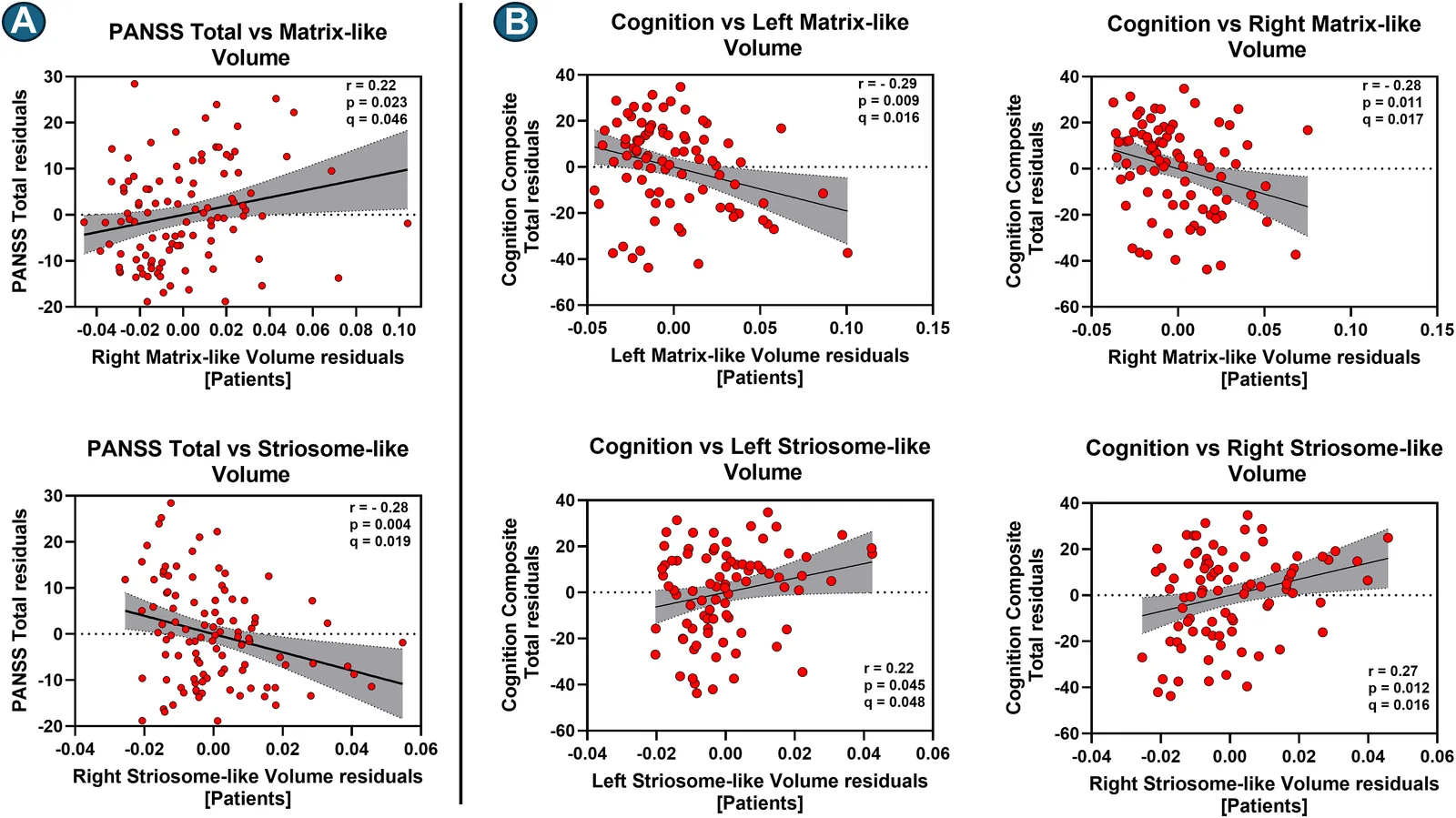
Clinical and cognitive correlates of striatal compartment volumes. **(A)** Partial regression plots showing associations between Positive and Negative Syndrome Scale (PANSS) total scores and striatal compartment volumes (matrix-like and striosome-like, left and right striatum). Higher PANSS total scores were associated with reduced striosome-like volume and to a lesser extent reduced matrix-like volume after controlling for age and sex. **(B)** Partial regression plots showing associations between global cognition (residualized composite score) and striatal compartment volumes. Better cognitive performance was associated with larger matrix-like volume and smaller striosome-like volume, controlling for age and sex. Bars represent standardized regression coefficients (βs) ± 95% CIs. Statistical significance (*p*) after false discovery rate correction is denoted by *q*.

Partial correlation analyses were performed between Positive and Negative Syndrome Scale (PANSS) total scores and striatal compartment volumes (left/right matrix- and striosome-like), controlling for age, sex, and eTIV, followed by Benjamini-Hochberg FDR correction. Across all patients, higher symptom severity was significantly associated with reduced right striosome-like volume (β = −198.11, *t* = −2.89, *p* = .0041, *q* = .0187) and greater right matrix-like volume (β = 94.49, *t* = 2.31, *r* = 0.22, *p* = .023, *q* = .046) (Figure 7A). Left-hemisphere effects were nonsignificant (*q*s > .1). When examined by subtype, these effects were weaker: In AP, associations between PANSS scores and right matrix-like volume were positive but nonsignificant (β = 177.11, *p* = .077, *q* = .31), and no striosome associations were observed (*q*s > .5). In NAP, PANSS total scores were inversely correlated with right striosome-like volume (β = −180.22, *t* = −2.38, *p* = .0199, *q* = .080), indicating a trend-level effect after correction. The results suggest that higher clinical symptom severity (PANSS total score) may be linked to reduced right striosome-like volume, consistent with compartment-specific vulnerability of the striosome system in psychosis, particularly within the right hemisphere.

### Cognitive Correlations With Striatal Compartment Volumes

Across the entire patient cohort, higher age-adjusted total cognitive performance was significantly associated with larger striosome-like and smaller matrix-like normalized volumes bilaterally. After controlling for age, sex, and total intracranial volume, cognition showed inverse associations with left and right matrix-like volumes (β = −190.4 ± 70.7, *p* = .0086 and β = −219.0 ± 84.1, *p* = .0110, respectively) and positive associations with left and right striosome-like volumes (β = 312.2 ± 153.3, *p* = .0451 and β = 349.6 ± 136.7, *p* = .0124). All 4 relationships remained significant following Benjamini-Hochberg FDR correction (*q*s < .05) (Figure 7B). In contrast, neither the AP subgroup nor the control group demonstrated significant relationships between cognitive performance and striatal compartment volumes. In the NAP group, effect directions were similar to those observed in the full patient sample, with the right striosome-like volume showing a trend-level positive association (*p* = .0233, *q* = .093). Together, these findings suggest that better cognitive functioning is linked to a relative expansion of striosome-like volume and a contraction of matrix-like volume in patients with early psychosis.

### Correlations Between Montgomery–Åsberg Depression Rating Scale Scores and Striatal Compartment Volumes

Across all patients, partial correlation analyses revealed no significant associations between Montgomery–Åsberg Depression Rating Scale (MADRS) total scores and normalized striatal compartment volumes after correction for multiple comparisons (Benjamini-Hochberg FDR, all *q*s > .05). In the combined patient group, associations were modest and negative in direction, indicating that higher depressive symptom severity tended to relate to slightly smaller striatal compartment volumes. Specifically, the correlations were *r* = −0.12 (*p* = .21, *q* = .48) for the left matrix, *r* = −0.10 (*p* = .28, *q* = .51) for the left striosome, *r* = −0.17 (*p* = .09, *q* = .36) for the right matrix, and *r* = −0.15 (*p* = .12, *q* = .40) for the right striosome. When examined separately, both the AP and NAP subgroups demonstrated similarly weak and nonsignificant associations. In the AP group, the largest effects were observed for the right matrix (*r* = −0.19, *p* = .17, *q* = .42) and right striosome (*r* = −0.14, *p* = .29, *q* = .55), while in the NAP group, the corresponding associations were *r* = −0.11 (*p* = .31, *q* = .57) and *r* = −0.08 (*p* = .40, *q* = .61), respectively. No effects reached even uncorrected significance (all *p*s > .05). Excluding participants with a MADRS score of 0 did not change the pattern of results. Collectively, these findings suggest that in patients with early psychosis, depressive symptom severity, as indexed by the MADRS, is not reliably associated with volumetric variation in either the matrix-like or striosome-like compartments of the striatal regions.

## Discussion

This study provides the first in vivo evidence of altered striosome-matrix organization in early psychosis. While overall compartment proportions were preserved, NAP showed matrix expansion, displacement of striosome centers, and increased compartment separation, particularly in the left striatum. These alterations were associated with symptom severity and cognition, highlighting their functional relevance.

### Striosome-Matrix Reorganization and Functional Implications in Psychosis

Our findings indicate altered striosome–matrix organization in early psychosis, with relative matrix expansion and increased imbalance, particularly in NAP. This reflects a redistribution between canonical compartments rather than the emergence of new tissue types. Such patterns align with models of disrupted corticostriatal integration between limbic and associative/sensorimotor inputs (17,36,37). Several mechanisms may underlie this matrix-dominant pattern, including altered cortical drive, compensatory network reorganization (38,39), or reduced striosomal contribution due to limbic input disruption or selective vulnerability. Given the smaller baseline volume of striosomes, proportional changes may further exaggerate matrix dominance at the imaging level. Additionally, displacement of striosomal centers may reflect altered limbic circuit organization, including orbitofrontal projections regulating dopaminergic function (26,40,41). Together, these findings support disrupted limbic-sensorimotor integration as a core feature of early psychosis.

Electrophysiological studies have shown that striosomal striatal projection neurons are more excitable than matrix neurons, with higher input resistance and lower firing thresholds (19,29,42), making them particularly sensitive to dopaminergic and stress-related perturbations (28,43). High-dopamine states preferentially engage striosomal circuits, while dopamine antagonists may help restore compartmental balance. Therefore, a relative matrix predominance, as was observed here, may reflect a shift away from limbic-striosomal regulation toward sensorimotor processing. This interpretation is consistent with postmortem evidence of reduced striosome density in schizophrenia (34) and similar compartmental imbalances reported in autism (23,24), suggesting a transdiagnostic mechanism affecting cognition, motivation, and behavior.

Lateralized effects, most prominent in the left striatum, may reflect hemispheric differences in corticostriatal and dopaminergic function. The left associative striatum is linked to executive control and language processes (44, 45, 46), which are often disrupted in psychosis (47,48), and prior studies report left-lateralized dopaminergic abnormalities in schizophrenia (49,50). Consistent with this, striosome-matrix alterations were more pronounced in NAP, with AP showing intermediate values. This pattern suggests closer links to core psychotic mechanisms, although smaller sample size and overlapping mood-related pathology may attenuate effects in AP. Such overlap is consistent with prior findings of striatal alterations in depression (51). Overall, these results support compartment-specific and lateralized contributions to psychosis heterogeneity, with greater disruption in nonaffective presentations.

### Clinical and Cognitive Correlations

Associations between striosome-matrix metrics and clinical measures highlight their functional relevance. Higher PANSS scores were linked to reduced striosome-like volume, particularly in the right striatum, consistent with the role of striosomes in dopaminergic regulation and stress responsivity (26,28). In contrast, better cognition was associated with greater striosome-like and reduced matrix-like volumes, suggesting that balanced compartmental organization supports corticostriatal integration (52). Similar compartmental alterations are observed across disorders, including matrix expansion in autism (23), mixed striosome-matrix shifts in depression (51), and matrix predominance with striosome loss in Parkinson’s disease (53). Together, these findings position the striosome-matrix system as a key axis linking dopaminergic dysfunction to symptom severity and cognitive variability, with early psychosis, particularly nonaffective forms, showing a shift toward matrix predominance. Although spatial organization metrics also showed significant group differences, we focused on volumetric measures in clinical association analyses due to their more direct interpretability as indices of compartmental balance. Spatial metrics may capture higher-order organizational features and could provide complementary insights, but their clinical relevance requires further investigation.

Our findings highlight the striosome-matrix system as a key axis linking dopaminergic and cortical dysfunction to clinical and cognitive variability. Early psychosis, particularly nonaffective subtypes, appears to be characterized by matrix predominance and reduced striosomal contribution to limbic-cognitive processing.

### Methodological Considerations and Limitations

This diffusion-based parcellation provides a noninvasive framework to study the striosome-matrix system but has limitations. First, voxel-level classification lacks histological precision, as each 2-mm voxel likely contains mixed tissue, introducing partial volume effects. Probabilistic tractography infers connectivity indirectly, cannot resolve synapses or directionality, and is susceptible to false positives, negatives, and interindividual variability. However, prior work has demonstrated high reproducibility (∼0.14% test-retest error) and consistency with known compartmental organization, supporting the anatomical specificity of this approach findings (54).

Second, the study’s sample size, particularly within diagnostic subgroups, was modest, constraining statistical power and potentially obscuring smaller or interaction effects. Future studies with larger, demographically balanced, and harmonized cohorts will be essential to confirm and extend these findings. Third, as the design was cross-sectional, causal inferences cannot be drawn regarding whether the observed abnormalities represent developmental vulnerability, early illness processes, or compensatory adaptation.

Fourth, although site harmonization via ComBat reduced scanner-related variance, subtle acquisition or preprocessing differences may persist. It is also important to note that while early-stage sampling minimizes chronicity-related confounds, illness duration and severity were not included, and effects of illness burden cannot be ruled out. Diffusion MRI cannot directly resolve the cellular or molecular features of striosome and matrix compartments. Multimodal approaches, including ultra-high-field MRI and molecular imaging, will be important for validation and mechanistic insight.

Despite these limitations, in vivo parcellation provides a unique bridge between preclinical models and human studies, enabling comparison of compartmental organization across psychosis subtypes. Quantifying deviations in matrix-striosome balance may help explain symptom heterogeneity and variability in treatment response.

Beyond structural characterization, compartment-like voxels provide a principled substrate for functional and effective connectivity analyses (54). Tasks engaging emotional salience, reward prediction, and motivational learning are expected to preferentially recruit striosome-associated territories, whereas motor execution, habit formation, and sensorimotor integration may more strongly engage matrix-associated regions. Therefore, applying compartment-informed connectivity and task-based analyses could elucidate how imbalances between limbic and sensorimotor striatal systems contribute to dopaminergic dysregulation, impaired cognitive control, and behavioral inflexibility in psychosis, thereby advancing a more mechanistic, circuit-level understanding of disease heterogeneity.

### Conclusions

Early NAP is associated with altered striosome-matrix architecture, including matrix predominance, displacement of striosomal centers, and increased compartmental imbalance. These patterns may reflect developmentally atypical organizations that become functionally relevant under dopaminergic and stress-related perturbations. Altered compartment balance may contribute to dysregulated dopamine signaling and cognitive impairment without requiring overt structural injury. Emerging evidence also highlights the striosome-matrix system as a therapeutic target, including compartment-specific effects on PDE10A and dopamine signaling (55). This noninvasive framework enables in vivo assessment of striatal compartment balance and may provide a circuit-level biomarker to guide patient stratification and treatment development. Future work integrating compartment-specific connectivity may further link these alterations to large-scale network dysfunction.

## Supplementary Material

Supplemental Methods and Table S1

Key Resources Table
