# Supplementary material for: Noninvasive Mapping of Striosome-Matrix Imbalance in Early Psychosis
Source: Biol Psychiatry Glob Open Sci. 2026 Jul 6;6(5):100785. doi: 10.1016/j.bpsgos.2026.100785 (PMC13488050; doi:10.1016/j.bpsgos.2026.100785)
Supplement: Supplementary file 1 [file mmc1.pdf]

SUPPLEMENTARY INFORMATION

Non-Invasive Mapping of Striosome–Matrix Imbalance in Early Psychosis

Weerasekera *et al.*

Supplementary Material

Imaging Data

Imaging was conducted at multiple sites across two cities: Brigham and Women's Hospital, McLean Hospital in Boston, Massachusetts, USA and the Indiana University School of Medicine in Indianapolis, Indiana USA. All facilities utilized a 3T Siemens Prisma MR scanner. Downloaded datasets included a high-resolution T1-weighted structural (MPRAGE, TR/TE/TI/flip angle= 2400ms/2.2ms/1000 ms/8°, number of excitations (NEX) = 1, number of slices=208, Slice voxel size=0.8 × 0.8 × 0.8 mm3, FOV=256 × 252 mm) scan and diffusion weighted (spin echo planar imaging (SE-EPI) sequences with a total of 92 different diffusion directions (repetition time/echo time = 3230/89.3 ms, NEX=1, 48 slices with slice thickness = 1.5 mm, field of view = 270 × 270 mm, and b-values = 3000/1500 s/mm2) MR data for each of the subjects.

Parcellating the striatum into striosome- and matrix-like compartments

Given the small and interdigitated nature of striosome and matrix compartments, we employed a connectivity-based parcellation approach rather than direct voxelwise anatomical segmentation. This method leverages known differences in corticostriatal connectivity to identify striosome- and matrix-enriched regions in vivo. Rigorous preprocessing, quality control, and exclusion of low-quality data were applied to ensure reliability. In addition, analyses were performed on normalized, compartment-level metrics to reduce sensitivity to voxel-level variability and partial volume effects. The observed spatial patterns were consistent with prior literature, supporting the validity of the approach.

Functionally, striosome and matrix compartments are differentially associated with behavioral and clinical outcomes. Striosomes are preferentially linked to limbic processing, reward valuation, and regulation of dopaminergic tone, and have been implicated in affective symptoms, stress responsiveness, and reinforcement learning. In contrast, the matrix is more strongly associated with sensorimotor and associative functions, including action selection, habit formation, and cognitive control. Accordingly, alterations in these compartments may differentially contribute to clinical phenotypes, with striosomal dysfunction linked to affective and motivational disturbances and matrix alterations associated with cognitive and behavioral impairments.

Five striosome-favoring regions, posterior orbitofrontal cortex, basal operculum, anterior insula, basolateral amygdala, and mediodorsal thalamus, were summed to generate a composite striosome-favoring target mask. Likewise five matrix-favoring regions, supplementary motor area, primary motor cortex, primary sensory cortex (*Brodmann areas 1–3*), combined ventrolateral thalamic nuclei (VLc and VPLo), and globus pallidus interna, were combined to form a composite matrix-favoring target mask.These composite masks were constructed to address two key observations from animal tract-tracing studies: (1) each cortical or thalamic region exhibits a projection bias toward one striatal compartment but contains a mixture of matrix- and striosome-projecting neurons (1, 2) and (2) compartmental specificity may be restricted to subregions of the striatum, with reduced selectivity elsewhere(3). Combining

connectivity across multiple compartment-biased regions mitigated potential off-target effects and enhanced signal specificity.

Tractography was performed in native diffusion space using FSL *probtrackx2* (version 6.0.4)(4) to generate compartment-specific connectivity maps for each hemi-striatum. Each hemi-striatum served as the seed region, with the ipsilateral composite matrix-favoring and striosome-favoring target masks defined as classification targets. Seed masks for the left and right striatum were derived from subject-specific anatomical segmentations of the caudate and putamen obtained from T1-weighted images (e.g., FreeSurfer), combined to form a whole-striatum mask for each hemisphere, and registered to diffusion space using boundary-based registration. To prevent cross-hemispheric contamination, a midline exclusion mask was applied in the sagittal plane, with left and right hemispheres processed independently. Streamline propagation was constrained using the following probtrackx2 parameters: curvature threshold = 0.2, step length = 0.5 mm, number of samples = 5,000 per voxel, maximum steps per sample = 2,000, path-length correction was applied to normalize connection probabilities for distance bias. For each voxel within a given hemi-striatum, tractographic connectivity probability (range: 0.0–1.0) was computed to both the matrix-favoring and striosome-favoring composite target masks, resulting in subject- and hemisphere-specific probability maps. Voxels were classified as matrix-like or striosome-like if the connection probability to one target exceeded 0.55 (i.e., ≥ 55% bias toward one compartment). The suffix "-like" reflects two inherent limitations of diffusion-based parcellation: first, human striosomes range from 0.5–1.25 mm in coronal diameter(5, 6), smaller than the 2 mm isotropic acquisition voxels, such that each voxel may contain a mixture of striosomal and matrix tissue; second, classification is based on structural connectivity patterns matching those observed in animal tract-tracing studies but does not constitute direct histological identification via immunohistochemical markers (e.g., mu-opioid receptor, calbindin), which remains the gold standard for striatal compartment delineation. This thresholding strategy enriched for voxels with predominant matrix or striosome-like connectivity profiles, minimizing off-target inclusion of mixed or non-specific tissue. Freesurfer's recon-all tool (version 7.1.1)(7) recon-all tool was used for segmentation of caudate and putamen volumes and to produce the estimated total intracranial volume (eTIV).

Volumetry properties of striatal compartments

Volumes of matrix-like and striosome-like parcellations were computed in native diffusion space at two probability thresholds: 0.95 (high confidence) and 0.55 (minimum classification threshold). Indeterminate voxels, defined as those with connection probabilities between 0.45 and 0.55, were quantified separately and excluded from compartment assignment. Compartment-like volumes were measured for the entire striatum and independently within the caudate and putamen subregions, using anatomically defined masks. Histogram analysis of the matrix-like probability distribution (range: 0.0–1.0) was conducted using FSL *fslstats* with 100 equal-width bins. As detailed in the Results section, theoretical modeling indicated that group comparisons restricted to the uppermost five bins (highest-probability voxels) of each distribution could isolate tissue-level mechanisms underlying volumetric abnormalities, such as selective loss or expansion of high-bias compartment tissue.

Exploratory diffusion metric extraction from striatal masks

To assess whether local diffusion properties could have influenced tractography-based compartment assignment, we performed an exploratory sensitivity analysis of diffusion tensor metrics within each

subject's matrix-like and striosome-like striatal masks. Our intent was not primarily to interpret FA or MD as definitive markers of striatal gray matter microstructure, but rather to determine whether systematic differences in diffusion properties between matrix-like and striosome-like voxels could bias streamline propagation or connectivity estimates independent of true matrix/striosome-like segregation. FA and MD maps were generated in native diffusion space, and mean values were extracted by averaging across all voxels within each mask. Because FA and MD are typically more sensitive to white matter microstructure and have limited biological specificity in gray matter structures such as the striatum, these measures were interpreted as exploratory sensitivity metrics to assess potential gross diffusion differences or partial-volume effects within the parcellated masks.

Defining striatal compartment masks for subsequent quantification steps

Probabilistic tractography can be biased by differences in target mask size. To ensure equitable quantification of matrix-like and striosome-like connectivity, equal-volume, compartment-specific striatal masks were generated for each hemisphere. Candidate voxels were drawn exclusively from high-bias regions (connection probability ≥ 0.55, ensuring compartment specificity) and selected in descending order of probability, beginning at 1.0. Voxel inclusion continued until a fixed volume threshold of 83 native-space voxels was reached per compartment. This procedure guaranteed that each compartment mask comprised an identical number of the most strongly biased voxels. The volume threshold of 83 voxels was derived from the MNI152 1 mm template hemi-striatum volume by: removing the central portion corresponding to the median $\pm$ 1.5 SD under a normal distribution, dividing the remaining volume equally between the two upper and lower tails. Thus, each 83-voxel mask represents the top 1.5 SD of the compartment-specific probability distribution, capturing the most discriminative tissue while controlling for mask size confounds.

Within each subject and hemisphere, matrix-like and striosome-like masks were constrained to identical volumes. Due to non-normal connectivity bias distributions in some participants, particularly those with strong bias toward one extra-striatal target class, 83 voxels meeting the ≥ 0.55, probability threshold could not always be identified for both compartments. In such cases, both compartment masks were truncated to N, the number of available high-bias voxels (≥ 0.55), in the probability map that had smaller volume, ensuring volume equivalence across compartments. The resulting mean mask volume was 82.5 voxels per compartment, with no significant difference between psychosis and control groups. These equal-volume masks were subsequently used as targets in a second round of probabilistic tractography to quantify compartment-specific connectivity.

Striosome–Matrix Bias Metrics

To quantify the relative abundance and spatial organization of striosome- and matrix-like compartments, voxelwise bias maps were aggregated into region-level measures within each striatal region and hemisphere. For each subject, the following metrics were extracted: total voxel count (N_region); number and percentage of voxels classified as striosome-like (N_strio_high, pct_strio_high) or matrix-like (N_matrix_high, pct_matrix_high); and the three-dimensional centers of mass (*COM_strio_x/y/z*, *COM_matrix_x/y/z*) for striosome- and matrix-like voxels. The Euclidean displacement between the two compartment centroids along each axis (*Delta_x, Delta_y, Delta_z*) indexed the degree of spatial

separation between striosome and matrix subregions. These metrics were computed separately for the left and right striatum.

ComBat Harmonization

To mitigate site-specific variance arising from scanner or acquisition differences, volumetric measures of the left and right striatal compartments (striosme and matrix) were harmonized across imaging sites using the ComBat framework(8, 9) implemented in *neuroHarmonize* (Python). The batch variable was defined as the scanner site, while biological covariates including age, sex, and eTIV were preserved in the model to retain meaningful anatomical variance. ComBat estimates and removes additive and multiplicative site effects from each feature, while adjusting data to a common distribution centered on the pooled mean across sites. This procedure effectively standardizes the mean and variance of each striatal measure across scanners, reducing inter-site bias while maintaining within-subject biological variability.

Statistical Analysis

Group differences in striatal compartment volumes were examined using analysis of covariance (ANCOVA) models implemented in *statsmodels* (Python 3.11). Four dependent variables were analyzed: left and right matrix-like and striosome-like striatal compartment volumes (0.95 thresholded). The primary between-subjects factor was Group (Control, Affective Psychosis, Non-Affective Psychosis), with age, sex, and estimated total intracranial volume (eTIV) included as covariates to control for demographic and global brain size effects. All variables were inspected for normality, outliers, and homogeneity of variance prior to analysis. When the omnibus ANCOVA revealed a significant main effect ($p < 0.05$), Tukey's Honest Significant Difference (HSD) post hoc tests were conducted on covariate-adjusted residuals to identify pairwise group differences. Multiple comparisons across the four regional models (L/R × Matrix/Striosome) were corrected using the Benjamini–Hochberg false discovery rate (FDR) procedure ($\alpha = 0.05$). Adjusted (covariate-controlled) means and 95% confidence intervals were extracted for visualization of directionality and effect magnitude.

Associations between clinical and cognitive measures and striatal compartment volumes were evaluated using partial correlation analyses based on ordinary least squares (OLS) regression in statsmodels. Dependent variables included the normalized volumes of the left and right matrix-like and striosome-like compartments. Clinical measures encompassed symptom severity (PANSS total), depressive symptoms (MADRS total), and cognitive performance. Cognitive function was assessed using an age-adjusted composite score derived from standardized neurocognitive tests (Total_Cognition_Composite_AgeAdjusted), reflecting overall performance across multiple cognitive domains. Cognitive impairment is a core feature of psychotic disorders and is strongly associated with functional outcomes; therefore, it was included as a clinically relevant dimension alongside symptom measures. Together, these measures capture complementary aspects of illness, including psychotic symptom severity, mood-related symptoms, and cognitive functioning. Independent variables (analyzed separately) included PANSS total score, MADRS total score, and the age-adjusted total cognition composite score. All models were adjusted for age, sex, and medication. Analyses were conducted first within the full patient sample and then repeated separately for the affective and non-affective psychosis

subgroups, as well as for healthy controls where applicable. For each model, standardized regression coefficients (β), t-values, and p-values were reported, and FDR correction (q-value, Benjamini–Hochberg) was applied within each analysis set (four regional comparisons per predictor). Residualized values of both structural and clinical measures (covariate-adjusted) were extracted for visualization, and residual scatter plots were generated for all significant or trend-level effects. Cohen's d was calculated to quantify group effect sizes (positive values indicating patient > control).

Supplementary Table 1

Variable	Included participants (N = 170)	Excluded datasets with available data (N = 19)	p-value
Total N	170	19	—
Healthy controls, n (%)	55 (32.4%)	2 (10.5%)	0.064
Patients, n (%)	115 (67.6%)	17 (89.5%)	0.064
Age, years, mean ± SD	Combined: 23.4 ± 3.9<br>Controls: 24.8 ± 4.0; Patients: 22.7 ± 3.7	Combined: 24.2 ± 3.2<br>Controls: 23.5 ± 3.6; Patients: 24.3 ± 3.3	0.306
Sex, M/F	106/64<br>Controls: 36/19; Patients: 70/45	12/6<br>Controls: 2/0; Patients: 10/6	0.802
Handedness, R/L	145/19<br>Controls: 46/9; Patients: 99/10	17/1<br>Controls: 2/0; Patients: 15/1	0.698
Full-scale IQ, mean ± SD	Combined: 106.6 ± 16.7<br>Controls: 115.9 ± 10.9; Patients: 102.2 ± 17.2	Combined: 104.5 ± 17.7<br>Controls: 116.2 ± 8.7; Patients: 103.1 ± 18.1	0.618
Affective psychosis, n (%)	27 (23.5% of patients)	1 (5.9% of patients)	0.121
Non-affective psychosis, n (%)	88 (76.5% of patients)	16 (94.1% of patients)	0.121
PANSS Total, mean ± SD	Combined: 80.7 ± 17.5<br>AF: 70.9 ± 15.4; NAF: 83.7 ± 17.1	Combined: 84.1 ± 10.6<br>AF: 68.7 ± 5.1; NAF: 85.1 ± 10.1	0.266
MADRS Total, mean ± SD	Combined: 11.6 ± 9.0<br>AF: 18.6 ± 9.8; NAF: 9.4 ± 7.5	Combined: 11.0 ± 4.8<br>AF: 19.2 ± 3.8; NAF: 10.5 ± 4.5	0.706
Lifetime antipsychotic exposure, months, mean ± SD	Combined: 14.1 ± 15.3<br>AF: 10.0 ± 16.0; NAF: 15.3 ± 14.9	Combined: 16.2 ± 12.7<br>AF: 8.0 ± 6.0; NAF: 16.7 ± 12.9	0.535
CPZ equivalent, mg/day, mean ± SD	Combined: 156.8 ± 223.0<br>AF: 48.1 ± 134.1; NAF: 190.2 ± 234.5	Combined: 162.9 ± 194.4<br>AF: 42.1 ± 112.1; NAF: 170.4 ± 198.2	0.908

Note. AF = affective psychosis; NAF = non-affective psychosis; PANSS = Positive and Negative Syndrome Scale; MADRS = Montgomery–Åsberg Depression Rating Scale; CPZ = chlorpromazine equivalent dose; SMD = standardized mean difference. Percentages for affective and non-affective psychosis are calculated among patients only. Sex and handedness sample sizes reflect available data. Because some excluded datasets lacked demographic or clinical variables by definition, sample sizes may vary across comparisons.

References:

1. Kincaid AE, Wilson CJ. Corticostriatal innervation of the patch and matrix in the rat neostriatum. J Comp Neurol. 1996;374(4):578-92.
2. Smith JB, Klug JR, Ross DL, Howard CD, Hollon NG, Ko VI, et al. Genetic-Based Dissection Unveils the Inputs and Outputs of Striatal Patch and Matrix Compartments. Neuron. 2016;91(5):1069-84.
3. Ragsdale CW, Jr., Graybiel AM. A simple ordering of neocortical areas established by the compartmental organization of their striatal projections. Proc Natl Acad Sci U S A. 1990;87(16):6196-9.
4. Behrens TE, Berg HJ, Jbabdi S, Rushworth MF, Woolrich MW. Probabilistic diffusion tractography with multiple fibre orientations: What can we gain? Neuroimage. 2007;34(1):144-55.
5. Graybiel AM, Ragsdale CW, Jr. Histochemically distinct compartments in the striatum of human, monkeys, and cat demonstrated by acetylthiocholinesterase staining. Proc Natl Acad Sci U S A. 1978;75(11):5723-6.

6. Holt DJ, Graybiel AM, Saper CB. Neurochemical architecture of the human striatum. J Comp Neurol. 1997;384(1):1-25.
7. Fischl B. FreeSurfer. Neuroimage. 2012;62(2):774-81.
8. Johnson WE, Li C, Rabinovic A. Adjusting batch effects in microarray expression data using empirical Bayes methods. Biostatistics. 2007;8(1):118-27.
9. Fortin JP, Cullen N, Sheline YI, Taylor WD, Aselcioglu I, Cook PA, et al. Harmonization of cortical thickness measurements across scanners and sites. Neuroimage. 2018;167:104-20.
